# The Glycan Hole Area of HIV-1 Envelope Trimers Contributes Prominently to the Induction of Autologous Neutralization

**DOI:** 10.1128/JVI.01552-21

**Published:** 2022-01-12

**Authors:** Anna Schorcht, Christopher A. Cottrell, Pavel Pugach, Rajesh P. Ringe, Alvin X. Han, Joel D. Allen, Tom L. G. M. van den Kerkhof, Gemma E. Seabright, Edith E. Schermer, Thomas J. Ketas, Judith A. Burger, Jelle van Schooten, Celia C. LaBranche, Gabriel Ozorowski, Natalia de Val, Daniel L. V. Bader, Hanneke Schuitemaker, Colin A. Russell, David C. Montefiori, Marit J. van Gils, Max Crispin, P. J. Klasse, Andrew B. Ward, John P. Moore, Rogier W. Sanders

**Affiliations:** a Department of Medical Microbiology and Infection Prevention, Amsterdam Infection & Immunity Institute (AI&AII), Amsterdam UMC, Location Meibergdreef, University of Amsterdam, Amsterdam, The Netherlands; b Department of Integrative Structural and Computational Biology, The Scripps Research Institute, La Jolla, California, USA; c Department of Microbiology and Immunology, Weill Cornell Medical College, New York, New York, USA; d Laboratory of Applied Evolutionary Biology, Department of Medical Microbiology and Infection Prevention, Amsterdam Infection & Immunity Institute (AI&AII), Amsterdam UMC, Location Meibergdreef, University of Amsterdam, Amsterdam, The Netherlands; e Centre for Biological Sciences and Institute for Life Sciences, University of Southampton, Southampton, England, United Kingdom; f Department of Experimental Immunology, Amsterdam Infection & Immunity Institute (AI&AII), Amsterdam UMC, Location Meibergdreef, University of Amsterdam, Amsterdam, The Netherlands; g Department of Surgery, Duke University Medical Center, Durham, North Carolina, USA; Emory University

**Keywords:** Env trimer, HIV-1, SOSIP, autologous neutralization, glycan shield, immunization, vaccine

## Abstract

The human immunodeficiency virus type 1 (HIV-1) trimeric envelope glycoprotein (Env) is heavily glycosylated, creating a dense glycan shield that protects the underlying peptidic surface from antibody recognition. The absence of conserved glycans, due to missing potential N-linked glycosylation sites (PNGS), can result in strain-specific, autologous neutralizing antibody (NAb) responses. Here, we sought to gain a deeper understanding of the autologous neutralization by introducing holes in the otherwise dense glycan shields of the AMC011 and AMC016 SOSIP trimers. Specifically, when we knocked out the N130 and N289 glycans, which are absent from the well-characterized B41 SOSIP trimer, we observed stronger autologous NAb responses. We also analyzed the highly variable NAb responses induced in rabbits by diverse SOSIP trimers from subtypes A, B, and C. Statistical analysis, using linear regression, revealed that the cumulative area exposed on a trimer by glycan holes correlates with the magnitude of the autologous NAb response.

**IMPORTANCE** Forty years after the first description of HIV-1, the search for a protective vaccine is still ongoing. The sole target for antibodies that can neutralize the virus are the trimeric envelope glycoproteins (Envs) located on the viral surface. The glycoprotein surface is covered with glycans that shield off the underlying protein components from recognition by the immune system. However, the Env trimers of some viral strains have holes in the glycan shield. Immunized animals developed antibodies against such glycan holes. These antibodies are generally strain specific. Here, we sought to gain a deeper understanding of what drives these specific immune responses. First, we show that strain-specific neutralizing antibody responses can be increased by creating artificial holes in the glycan shield. Second, when studying a diverse set of Env trimers with different characteristics, we found that the surface area of the glycan holes contributes prominently to the induction of strain-specific neutralizing antibodies.

## INTRODUCTION

The human immunodeficiency virus type 1 (HIV-1) trimeric envelope glycoprotein (Env) is located on the surface of virus particles and is the target of neutralizing antibodies (NAbs), which are produced during infection. Accordingly, the Env trimer is central to vaccine development strategies aimed at inducing NAbs (1). The assembled Env trimer consists of three heterodimers, each formed by a gp41 and gp120 subunit. Vaccines based on Env, such as native-like SOSIP trimers, induced autologous and, sporadically, heterologous tier 2 NAbs in animals (2–5). Two major complications to the induction of potent and consistent neutralization breadth are the extreme diversity of HIV-1 Env and its extensive glycosylation (6–8). The trimer contains around 90 potential N-linked glycosylation sites (PNGS), which account for approximately half of the molecular mass of the external domains of the Env trimer (9). Differences in the number and precise locations of these glycans contribute to the overall variation in Env proteins. During trimer synthesis in the endoplasmic reticulum (ER), N-linked glycans can be attached to a PNGS that is defined by the motifs asparagine-x-threonine (NxT) or asparagine-x-serine (NxS), where x can be any amino acid except proline (10, 11). Glycans are attached to Env as oligomannose-type glycans, of which some are further processed in the Golgi compartment, while others remain underprocessed, particularly ones that form a cluster on the gp120 outer domain and that are located at the trimer apex (12–15).

The densely packed glycans on the trimer surface shield the underlying peptidic surface from recognition by the immune system. Nevertheless, the glycan shield is not impenetrable. First, although N-linked glycans are host cell derived and generally poorly immunogenic, they can contribute to multiple protein/glycan composite epitopes for broadly neutralizing antibodies (bNAbs) (7, 16). Second, the glycan shields often have holes created by the absence of one or more PNGS that are typically well conserved (i.e., present in >50% of HIV-1 group M strains). Glycan holes tend to be immunogenic and can induce strain-specific, autologous NAb responses (4, 17–19). Thus, knocking-out selected glycans on Env trimers increases the autologous NAb response, which is directed to the newly created holes (19, 20). As HIV-1 isolates with a complete glycan shield on Env might induce bNAbs more readily than ones with holes, glycan-dense trimers have been designed accordingly (3, 21–23).

To date, studies on how glycan holes influence SOSIP trimer immunogenicity have involved only trimers from a few genotypes. In this study, we sought to increase our understanding of this relationship by introducing artificial holes in the naturally dense glycan shields on trimers from the subtype B strains AMC011 and AMC016. In addition, we analyzed how well a large panel of SOSIP trimers with different glycan shield characteristics could induce autologous NAbs. Factors that could contribute to the induction of autologous NAbs were defined and assessed with a linear regression analysis. This analysis pointed at glycan hole area as a major driver for the induction of autologous NAb responses. Since multiple SOSIP trimers are moving into clinical phase testing (24), it is important to increase our understanding of the relationship between glycan holes and autologous NAb responses. Defining immunodominant glycan holes on Env trimers could facilitate the redesign of these trimers, as holes can be opened or closed as desired (19).

## RESULTS

### The deletion of conserved PNGS alters the glycan shield of AMC011 and AMC016 trimers.

To assess the impact on autologous neutralization when glycan holes are introduced into an otherwise complete glycan shield, we worked with two subtype B SOSIP trimers. The AMC011 SOSIP trimer, derived from a participant of the Amsterdam Cohort Studies (ACS), has a complete glycan shield; i.e., all the conserved PNGS, defined as being present in >50% of HIV-1 group M viruses, are present in the AMC011 sequence (3). We have reported previously that AMC011 SOSIP trimers induced autologous NAbs weakly and inconsistently in immunized rabbits (3). The second subtype B trimer, AMC016 SOSIP, which has not been described previously, also has an apparently complete glycan shield as defined by the presence of all conserved PNGS.

**FIG 1:**
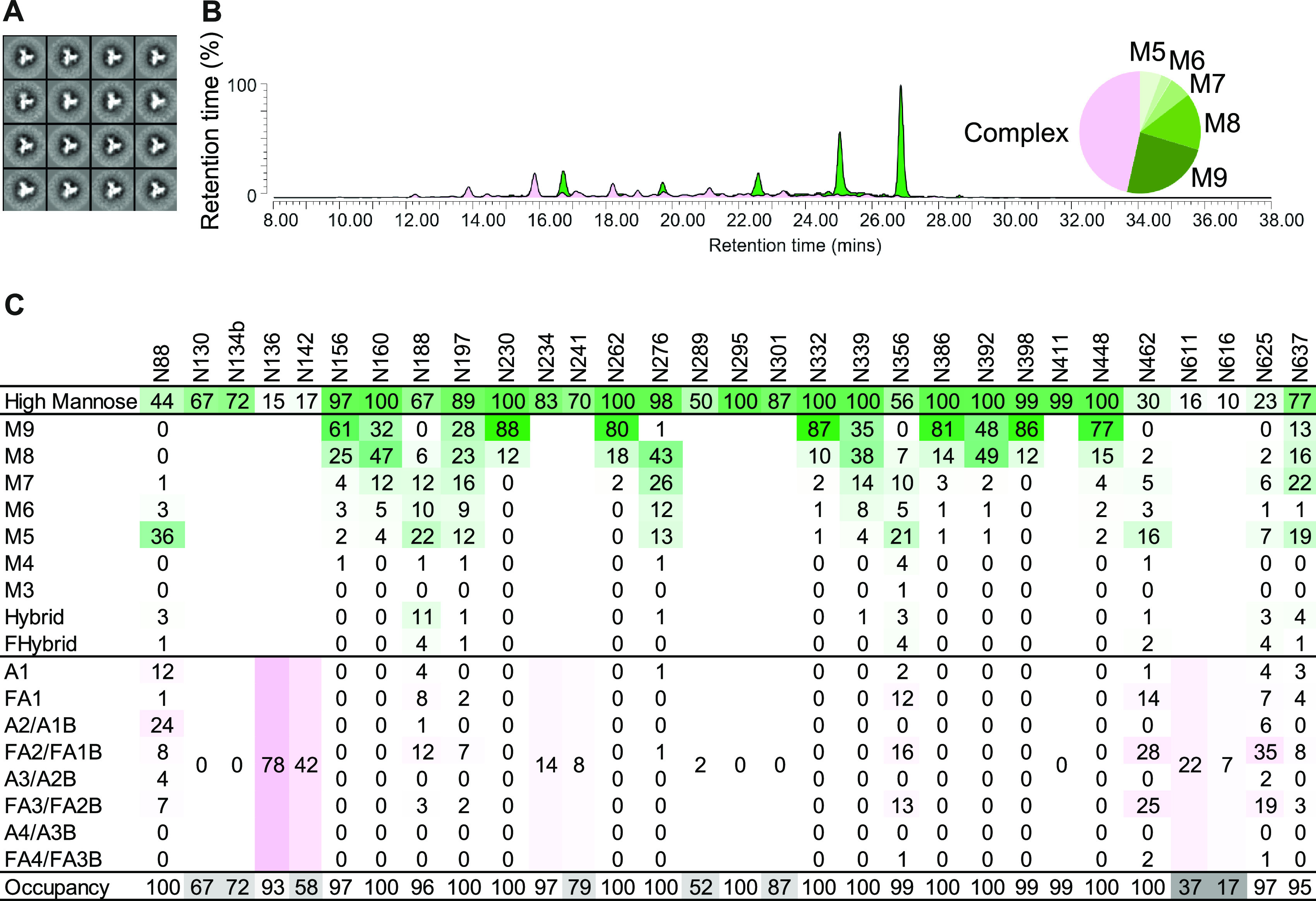
Characterization of the AMC016 SOSIP.v4.2 trimer. (A) 2D class averages derived from negative stain EM (NS-EM). (B) HILIC-UPLC analysis. Depicted in green are oligomannose/hybrid-type glycans and in magenta are fully processed complex-type glycans. (C) Site-specific glycan composition and occupancy using LC-MS on all 29 PNGSs. The color code is the same as in B. The oligomannose/hybrid-type glycans are categorized according to the number of mannose residues and the presence or absence of fucose, respectively. Fully processed complex-type glycans are arranged by the number of processed antenna and the presence of absence of fucose. The percentage of PNGSs that are <90% occupied are indicated in gray.

The AMC016 *env* sequence was obtained at 7 months postseroconversion from an ACS-participant that did not develop bNAbs. Stabilizing mutations were introduced to the gp140 sequence to create AMC016 SOSIP.v4.2 (2). The PGT145-purified protein was analyzed with negative-stain electron microscopy (NS-EM) and differential scanning calorimetry (DSC). The protein had a native-like trimer morphology (100%), and its midpoint of thermal denaturation (*T*_m_) was 63°C (Fig. 1A and Table 1). The AMC016 trimer structure was solved by cryo-EM and is presented below.

**TABLE 1:** Biophysical properties of AMC011 and AMC016 trimers

Property	Test	Parameter	Analyzed trimers			
			AMC011 SOSIP.v5.2		AMC016 SOSIP.v4.2	
			Parental	Mutant	Parental	Mutant
Production^*a*^		Yield (mg/L)	2.1^*b*^	1.0	2.0	1.5
Thermostability	DSC	Two-state model (*T*_m_ [°C])	67^*b*^	64^*c*^	63^*c*^	63^*c*^
Morphology	NS-EM	Native-like trimers (%)	100^*b*^	88	100	78
Glycan composition	HILIC-UPLC	Man_5-9_ (%)	58.2^*b*^^,^^*d*^	50.0^*b*^	53.4^*b*^	57.6^*b*^
		Man_9_ (%)	23.0^*b*^^,^^*d*^	16.5^*b*^	23.9^*b*^	26.4^*b*^

a Results were obtained from 293F cell-expressed and PGT145-purified SOSIP trimers.

b From reference 3.

c Results were obtained with D7324-tagged proteins.

d Quantified without Endo H digestion.

To get a better understanding of the glycans present on the AMC016 SOSIP.v4.2 trimer, we first analyzed the overall glycan composition, using hydrophilic interaction chromatography-ultraperformance liquid chromatography (HILIC-UPLC) (Fig. 1B and Table 1). The majority of glycans are oligomannose type (53.4% are Man_5-9_), of which nearly half (23.9%) are Man_9_GlcNac_2_ (here referred to as Man_9_). For comparison, the AMC011 SOSIP.v5.2 trimer has a slightly higher proportion of oligomannose-type glycans (58.2%), whereas the Man_9_ content was nearly the same (23.0%) (3).

Second, the site-specific glycan composition and occupancy of all 29 conserved PNGS on the AMC016 trimer were assessed by liquid chromatography-mass spectrometry (LC-MS). Most of the PNGS are dominated by oligomannose glycans (Fig. 1C, green), but the N136, N142, N462, and N625 sites contain predominantly complex glycans (Fig. 1C, pink). The high number of oligomannose glycans might reflect how a dense glycan shield restricts mannosidase access to individual sites (16). The majority of PNGSa are fully occupied, or almost so (>90%), but there is lower occupancy of the N130, N134b, N142, N241, N289, N301, N611, and N616 sites (67%, 72%, 58%, 79%, 52%, 87%, 37%, and 17% occupied, respectively) (Fig. 1C, gray). Overall, the AMC011 and AMC016 SOSIP trimers were similar in respect of the number and location of PNGS (3). Thus, on the AMC011 trimer, the PNGS are mostly occupied by oligomannose glycans, but with mostly complex glycans at the N88, N141c, N355, N461, and N625 sites (3). The N141, N241, N611, N616, and N637 sites are <90% occupied (22%, 86%, 8%, 3%, and 85% occupied, respectively). The composition and occupancy of N289, N392, and N396 glycans on the AMC011 trimer could not be resolved (3).

**FIG 2:**
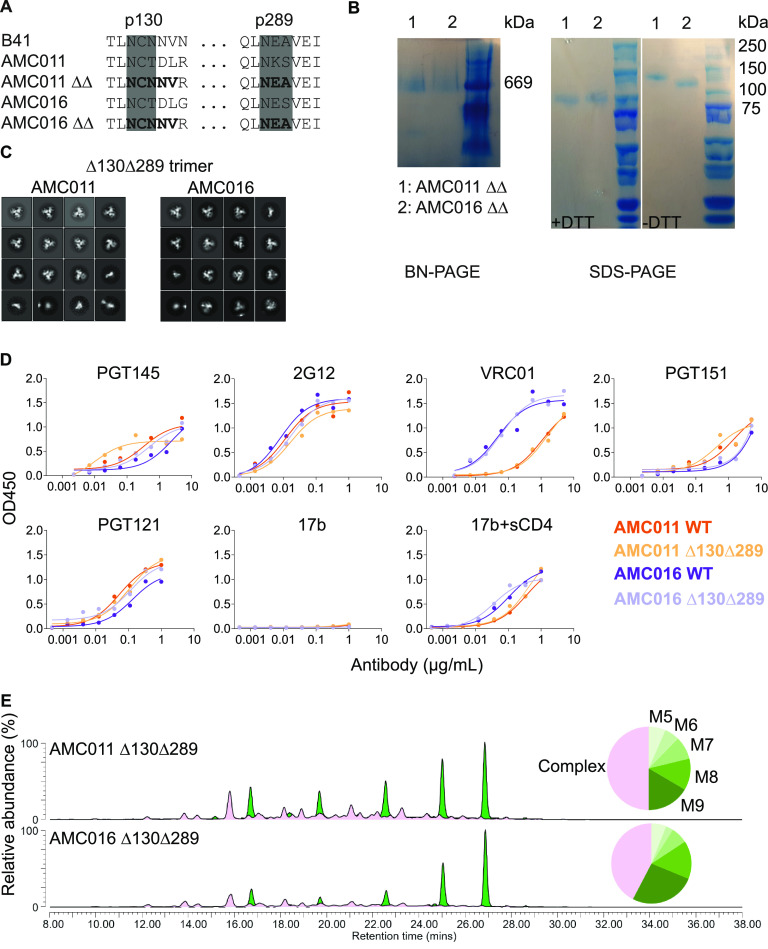
Characterization of the glycan mutant trimers AMC011 SOSIP.v5.2 Δ130Δ289 and AMC016 SOSIP.v4.2 Δ130Δ289. (A) Sequences of the AMC011 and AMC016 wild type (WT) and glycan mutants (indicated as ΔΔ) at positions 130 and 289 (HXB2 nomenclature) compared with B41. (B) BN-PAGE analysis (left) and SDS-PAGE analyses (right) under reducing (+dithiothreitol [DTT]) and nonreducing conditions (−DTT). The glycan mutant trimers are indicated as ΔΔ. (C) NS-EM analysis of the AMC011 Δ130Δ289 and AMC016 Δ130Δ289 trimers. (D) An ELISA was conducted to compare the antigenicity of the parental and glycan mutant SOSIP trimers using a panel of bNAbs and non-neutralizing antibody 17b. (E) Glycan composition of the AMC011 Δ130Δ289 and AMC016 Δ130Δ289 trimers, analyzed by HILIC-UPLC. Green, oligomannose/hybrid-type glycans; magenta, fully processed complex-type glycans. See Table 1 for details.

The two subtype B trimers AMC011 and AMC016 have a complete glycan shield, as judged by the presence of all conserved PNGS and hence are suitable for studying the impact of glycan holes on autologous neutralization. When designing the holes in the glycan shields, we used the subtype B B41 SOSIP trimer as a frame of reference for the PNGS we deleted. This trimer lacks two conserved glycans, namely, N130 and N289 (18, 25). Rabbits immunized with B41 SOSIP trimers developed strong NAb responses against the autologous virus that were directed against the N289 glycan hole, a finding confirmed by the cloning of monoclonal antibodies (18, 26). The N130 glycan hole, located at the trimer apex, was reported to have no or only a minor effect on the induction of autologous NAbs (18). We knocked out both the conserved PNGS at N130 and N289 to create the Δ130Δ289 trimers (Fig. 2A). The goal was to see whether the new holes would be immunogenic for autologous NAbs in the context of the AMC011 and AMC016 SOSIP trimers. In both cases, we mimicked the amino acid composition at the bottom of the corresponding holes on the B41 trimer. Thus, the NCT motif at N130 was altered to NCN and the next two residues DL were changed to their B41 counterparts, namely, NV (i.e., NCTDL to NCNNV). Similarly, the PNGS at N289 were changed from NKS to NEA for AMC011 SOSIP and NES to NEA for AMC016 SOSIP.

The resulting AMC011 and AMC016 SOSIP Δ130Δ289 trimers were expressed, affinity purified using PGT145, and characterized. The purified proteins were trimers (Fig. 2B, BN-PAGE) that were fully cleaved between gp120 and gp41 (Fig. 2B, SDS-PAGE). These yields were slightly lower than those for the parental trimer (1.0 mg/liter versus 2.1 mg/liter and 1.5 mg/liter versus 2.0 mg/liter for the AMC011 and AMC016 trimers, respectively) (Table 1). NS-EM showed that both trimers were predominantly, although not completely, in a native-like structure (88% for AMC011 SOSIP Δ130Δ289 and 78% for AMC016 SOSIP Δ130Δ289) (Fig. 2C and Table 1). These levels were lower than those seen with the parental AMC011 and AMC016 SOSIP trimers, which had fully native-like structures (100% in both cases) (Table 1) (3). In a DSC analysis, the *T*_m_ values for the AMC011 parental and Δ130Δ289 trimers were 67°C and 64°C, respectively, while the corresponding values for the AMC016 parental and Δ130Δ289 trimers were both 63°C (Table 1). The glycan-deleted AMC011 and AMC016 trimers had similar antigenicity profiles to the corresponding parental trimers when probed in an enzyme-linked immunosorbent assay (ELISA) using a panel of bNAbs and the non-neutralizing antibody 17b, with and without soluble CD4 (Fig. 2D).

We used the HILIC-UPLC method to study how the glycan deletions affected the overall composition of the glycan shield and to compare the mutant trimers with their parental counterpart. Knocking out the N130 and N289 sites slightly decreased the oligomannose glycan content of the AMC011 trimer (58.2% for parental versus 50% for the glycan mutant) (Fig. 2E and Table 1) (3). The largest decrease was observed for Man_9_ (23% versus 16.5%). Deleting both glycans had the opposite effect on the AMC016 trimer in that the oligomannose glycan content increased slightly (53.4% for parental versus to 57.6% for the glycan mutant), and the largest increase was again for Man_9_ (23.9% versus 26.4%) (Fig. 2E and Table 1).

**FIG 3:**
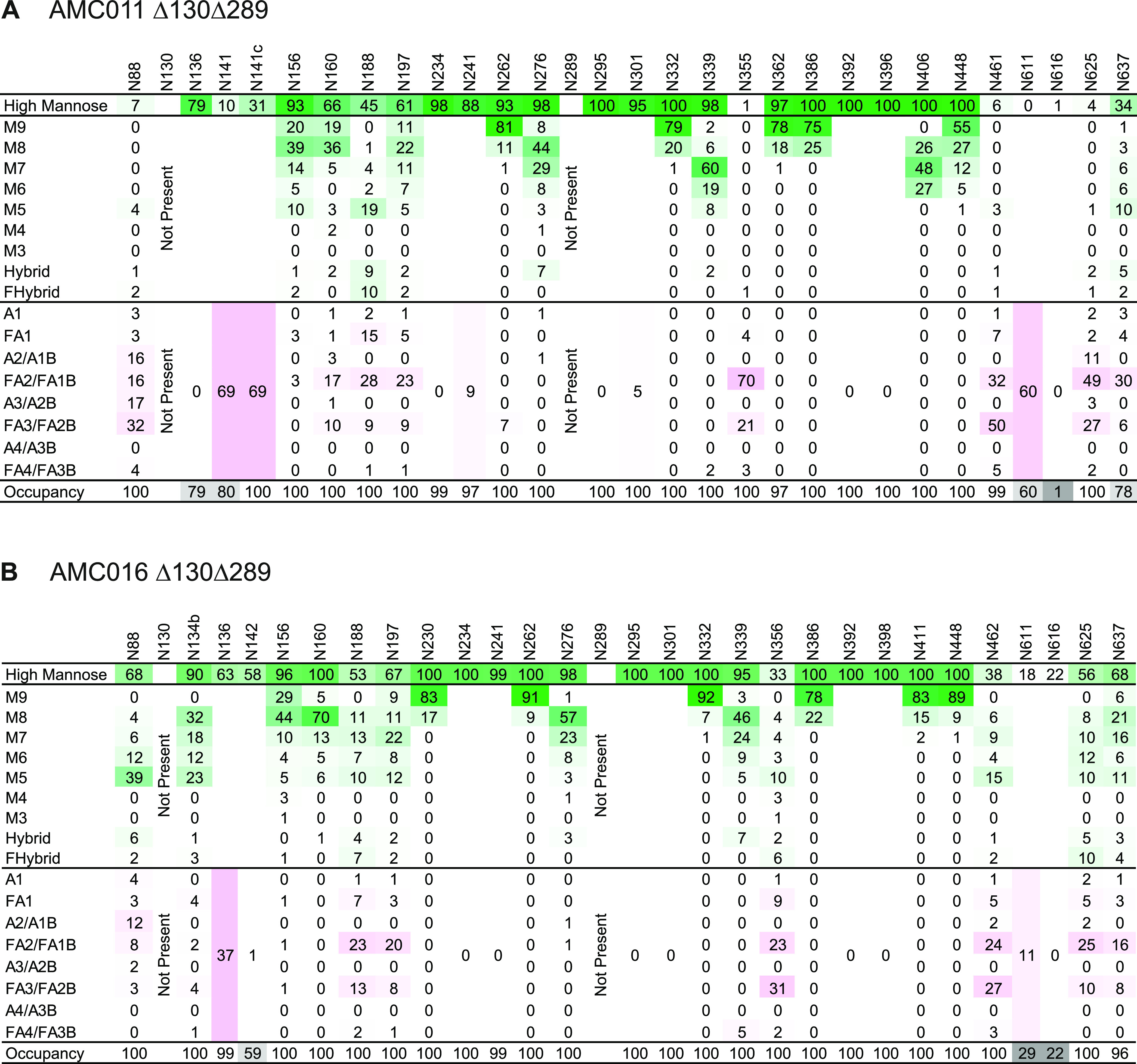
Site-specific glycan composition and occupancy of the AMC011 and AMC016 trimers from which N130 and N289 were deleted. (A) AMC011 SOSIP.v5.2 Δ130Δ289. (B) AMC016 SOSIP.v4.2 Δ130Δ289 D7324-tagged. The data were obtained by LC-MS on all PNGS. The color coding is the same as that used in Fig. 1.

The mutant trimers were also studied by LC-MS to obtain information on the site-specific glycan composition and occupancy. The majority of the 28 PNGS analyzed on the AMC011 Δ130Δ289 trimer were oligomannose-type glycans (Fig. 3A). However, the N88, N141, N141C, N188, N355, N461, N611, and N625 sites had >50% processed, complex-type glycans. Most of the PNGS were fully occupied; sites that were occupied to <90% were N136, N141, N611, N616, and N637 (79%, 80%, 60%, 1%, and 78%, respectively). Similarly, the 29 PNGS on the AMC016 Δ130Δ289 trimer were occupied mostly by oligomannose-type glycans, although >50% of the glycans on the N356 and N462 sites were complex (Fig. 3B). Again, PNGS occupancy was high, with the exception of N142, N611, and N616 (59%, 29%, and 22% occupied, respectively).

**FIG 4:**
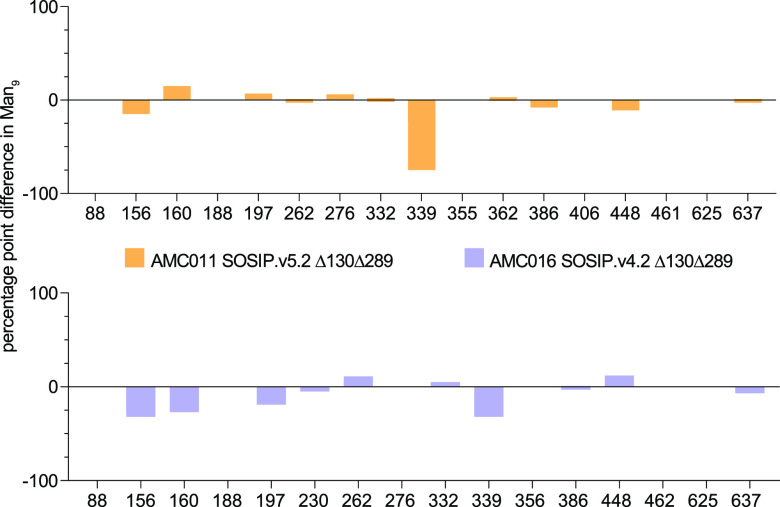
Percentage point difference in Man_9_ content at each site. Differences were calculated at sites where Man_9_ was resolved (% Man_9_ mutant trimer − % Man_9_ parental trimer) and indicated on the *y* axis. The PNGSs are listed on the *x* axis. Yellow, AMC011 SOSIP Δ130Δ289; lilac, AMC016 SOSIP Δ130Δ289 D7324-tagged. Glycan composition data on individual PNGSs were obtained by LC-MS (for individual values see Fig. 3).

A comparison of the glycan mutants with the corresponding parental trimers showed that knocking out the N130 and N289 glycans altered the Man_9_ content at a few specific PNGS (Fig. 4). The percentage point (pp) difference was calculated (% Man_9_ mutant trimer − % Man_9_ parental trimer) for sites where Man_9_ was resolved (see Fig. 3) (16). For AMC011 Δ130Δ289, the glycan knockout resulted in a substantial decrease in Man_9_ at the N339 site (75 pp decrease) (Fig. 4, yellow), which is adjacent to where the N289 glycan would be located. This outcome is consistent with previous observations that knocking out one glycan site can increase mannosidase access to nearby glycans but not more distant ones (16). In the case of the AMC016 Δ130Δ289 trimer, the glycan knockout also decreased the Man_9_ content of N339 (32 pp drop) (Fig. 4, lilac). In addition, the Man_9_ content decreased at sites N156, N160, and N197 (32, 27, and 19 pp decrease, respectively), which are located on the trimer apex and in close proximity to the N130 glycan hole.

Knocking out the N130 and N289 glycans had moderate impact on PNGS occupancy (see Fig. 1C and Fig. 3). Thus, N141 and N611 occupancy on the AMC011 Δ130Δ289 trimer increased by 58 and 52 pp, respectively, reaching 80% and 60%. In contrast, occupancy decreased for N136 by 21 pp, reaching 79%. For the AMC016 Δ130Δ289, N134b, N241, and N301 occupancy increased by 28, 20, and 13 pp, respectively, reaching 99% to 100%.

In summary, we produced two stable, native-like Δ130Δ289 trimers based on the subtype B isolates AMC011 and AMC016. Both mutant trimers have comparable biophysical and biochemical properties to their parental counterpart, although the percentages in the native-like form were slightly reduced. Knocking out the N130 and N289 PNGS had a localized impact on the composition and occupancy of a few neighboring glycans but only subtle effects elsewhere. The mutant trimers resemble the B41 SOSIP trimer in respect to the number and position of holes in their glycan shields, which are otherwise complete.

### The introduction of glycan holes promotes autologous NAb responses.

**FIG 5:**
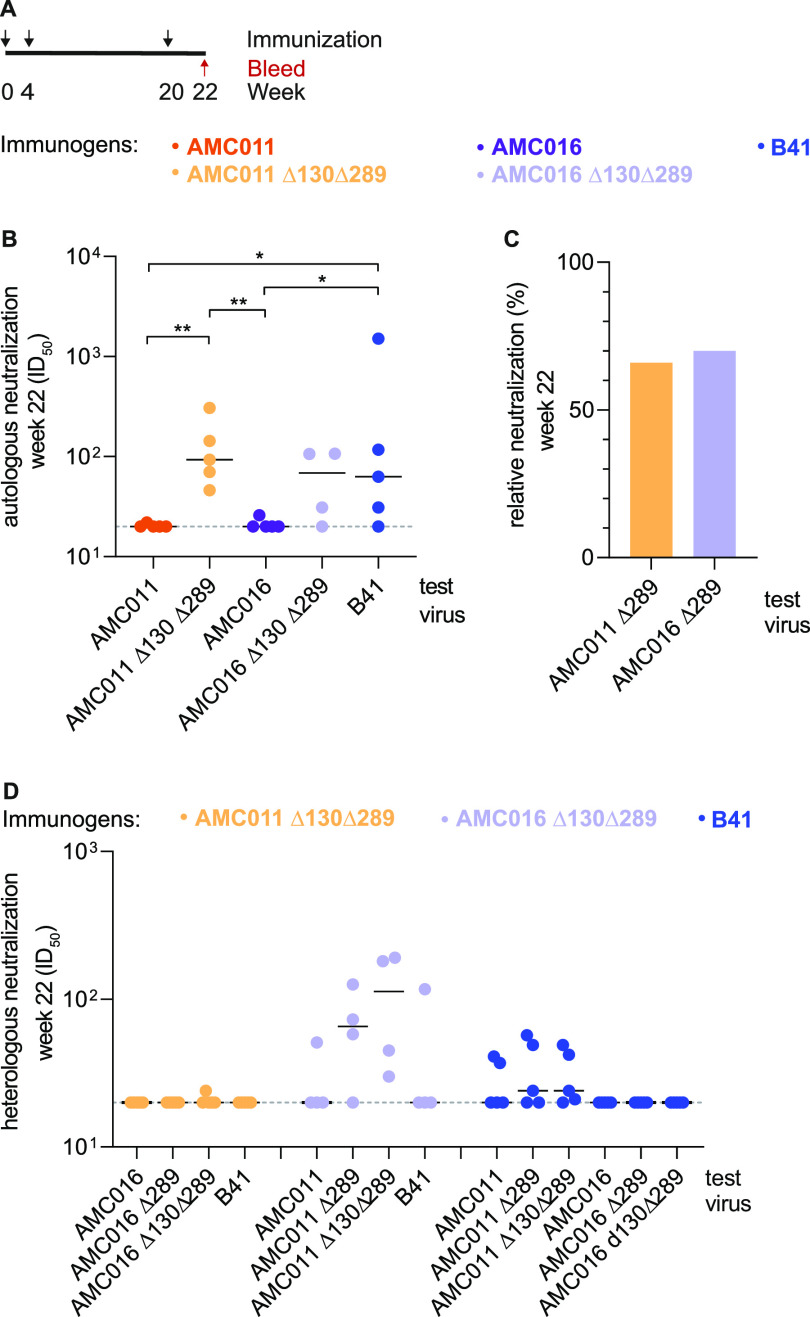
Immunogenicity of rabbits immunized with the parental AMC011 and AMC016 trimers and the AMC011 Δ130Δ289 and AMC016 Δ130Δ289 trimer variants, formulated with GLA-LSQ adjuvant. (A) Immunization schedule. Rabbits were immunized at week 0, 4, and 20, which are indicated with black arrows. Sera from the week 22 bleed was analyzed (red arrow). The groups are indicated and color-coded according to the immunogen they received. B41 trimer-immunized animals from the same study were included for comparison (19). Statistically significant differences are indicated. The data were analyzed with a two-tailed Mann-Whitney *U* test. (B) Autologous neutralization titers (ID_50_). (C) Relative neutralization of the AMC011 Δ289 and AMC016 Δ289 virus variants, based on the median ID_50_ values. Autologous neutralization of the Δ130Δ289 virus variants was defined as 100%, and the titers against the Δ289 variants were compared with this benchmark. (D) Heterologous neutralization of the AMC011Δ289 and Δ130Δ289 viruses, the AMC016 Δ289 and Δ130Δ289 viruses, and B41. (B, C, and D) The test viruses are indicated on the *x* axis. (B and D) The median ID_50_ per group is indicated by the horizontal black line; the dashed line represents the lower assay cutoff ID_50_ value of 20. Week 22 serum from rabbit 2295 (AMC016 Δ130Δ289 trimer group) interfered with MLV infection and was therefore excluded from further analysis. All individual ID_50_ values can be found in Supplemental file 1.

To test the impact of glycan holes on the induction of autologous NAbs, rabbits were immunized with the AMC011 Δ130Δ289 and AMC016 Δ130Δ289 trimers, formulated with GLA-LSQ adjuvant, as well as their parental counterparts. In the same study, a group of rabbits received the B41 trimer; data from this group, but not the other four, have been described previously (19). To allow comparability, sera from the B41 group were reanalyzed in the same assays as those for the AMC011 and AMC016 groups. Sera from week 22, which was 2 weeks after the third immunization, were assessed for autologous neutralization against the sequence-matched virus (Fig. 5A; see Supplemental file 1 in the supplemental material). Murine leukemia virus (MLV) served as a negative control. One serum sample in the AMC016 Δ130Δ289 group was excluded from analysis because it interfered with MLV infection (see figure legend and Supplemental file 1). Note that the GLA-LSQ adjuvant used in this study is now known to support SOSIP trimer immunogenicity inefficiently, which accounts for the lower autologous NAb titers against B41 and AMC011 than we reported previously (2, 3, 19).

The parental AMC011 and AMC016 SOSIP trimers induced NAbs against the autologous viruses weakly and inconsistently; the median 50% inhibitory dilution (ID_50_) values of 20 were not greater than the assay sensitivity limit (Fig. 5B; for individual values see Supplemental file 1). The glycan mutant trimers induced higher autologous titers (median ID_50_ of 93 and 69 for AMC011 Δ130Δ289 and AMC016 Δ130Δ289, respectively; *P* = 0.0079 for comparison of AMC011 SOSIP versus AMC011 Δ130Δ289 SOSIP; not significant for AMC016 SOSIP versus AMC016 Δ130Δ289 SOSIP).

The autologous NAb titers for the AMC011 and AMC016 parental trimer groups (median ID_50_ of <20 for both groups) were also significantly lower than those for the B41 trimer group (median ID_50_ of 63 for B41 immunized animals; *P* = 0.0476 versus either AMC011 or AMC016 parental trimers). The B41 autologous NAb titers were, however, similar to those induced by the AMC011 Δ130Δ289 and AMC016 Δ130Δ289 trimers (Fig. 5B). The NAbs induced by the AMC011 Δ130Δ289 and AMC016 Δ130Δ289 trimers did not neutralize the parental AMC011 and AMC016 viruses, implying that they were indeed targeting the glycan holes (median ID_50_ of 20 and 26, respectively) (see Supplemental file 1).

The analyses were then extended to include the AMC011 and AMC016 Δ289 virus variants. For each genotype, the median ID_50_ value was defined as 100% for the Δ130Δ289 virus. Compared with this benchmark, the titers against the Δ289 virus were 66% and 70% for AMC011 Δ130Δ289- and AMC016 Δ130Δ289-immunized animals, respectively (analysis based on Fig. 5C). Thus, the data imply that the N289 glycan hole plays a major role in the induction of autologous NAbs.

In a further analysis, we found that sera from the AMC016 Δ130Δ289 trimer-immunized rabbits cross-neutralized the AMC011 Δ289 and Δ130Δ289 virus variants (median ID_50_ values of 66 and 113, respectively) (Fig. 5D and Supplemental file 1). But, the AMC011 Δ130Δ289- and B41-trimer sera did not cross-neutralize the AMC016 glycan-deleted virus variants (the median ID_50_ values of 20 in all cases were not greater than the assay detection limit). The AMC011 and AMC016 Δ130Δ289 immunization sera also did not neutralize the B41 virus (median ID_50_ values of 20). Thus, the induction of cross-reactive NAb responses, even against very similar glycan holes, remains challenging.

### Diverse SOSIP trimers induce different levels of autologous neutralization.

Based on the findings outlined above, we hypothesized that the number of missing PNGS influences the ability of trimers to induce autologous NAbs against glycan holes. To test this hypothesis, we analyzed a large panel of sera from rabbits immunized with 1 of 11 SOSIP trimers derived from different subtypes, with various numbers of missing conserved PNGS (0 to 4) (see Supplemental file 2 in the supplemental material). Specifically, we assessed the ability of the various trimers, formulated in Iscomatrix adjuvant, to induce autologous NAbs.

The trimer and virus genotypes were as follows (see Supplemental file 2 for details): BG505 (subtype A); AMC008, AMC009, AMC011, AMC016, AMC018, B41, and TRJO (subtype B); and ZM197M, DU422, and CZA97.012 (subtype C). The AMC011, AMC016, AMC011 Δ130Δ289, AMC016 Δ130Δ289, and B41 trimer immunogenicity data that are described in Fig. 5 were not included in this analysis as the adjuvant was different. Instead, published immunogenicity data of rabbits immunized with the AMC011 and B41 trimers in Iscomatrix adjuvant were included in the analysis presented in the figures (Fig. 5 and Fig. 8) (2, 3). Published data on autologous NAbs responses to the BG505, AMC008, AMC009, and ZM197M trimers were also used (2, 3, 27). The autologous NAb titers induced by the AMC016, AMC018, TRJO, DU422, and CZA97.012 trimers, with Iscomatrix adjuvant, have not been reported elsewhere.

**FIG 6:**
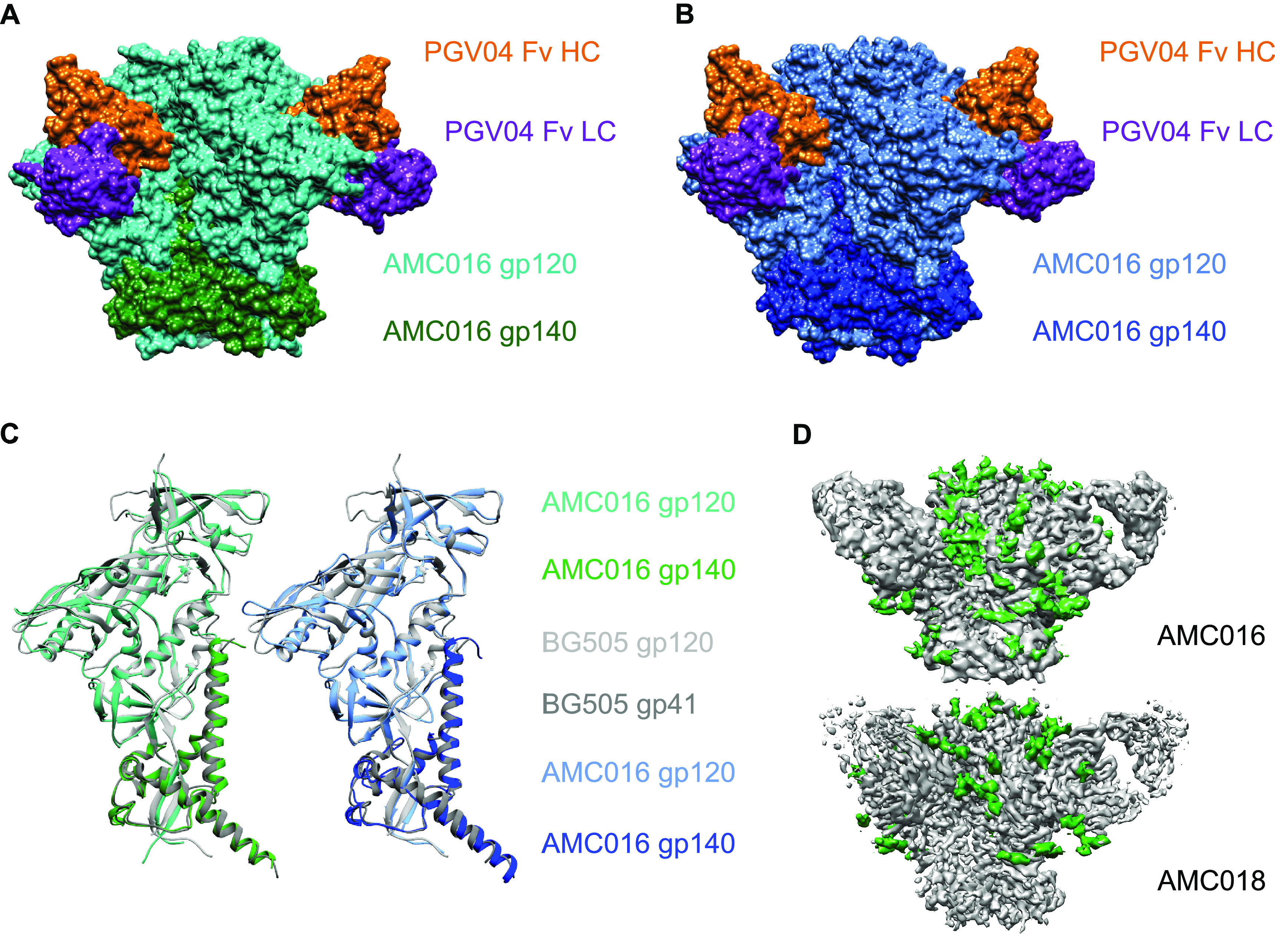
Cryo-EM structures of the AMC016 and AMC018 trimers. Molecular surface representation of AMC016 SOSIP.v4.2 + PGV04 Fab (A) and AMC018 SOSIP.v4.2 + PGV04 Fab (B). (C) Structural overlays of AMC016 SOSIP.v4.2 and AMC018 SOSIP.v4.2 with BG505 SOSIP.664. (D) EM density maps for AMC016 SOSIP.v4.2 + PGV04 Fab and AMC018 SOSIP.v4.2 + PGV04 Fab with density corresponding to N-linked glycans in green. Data collection parameters are provided in Table 2.

**FIG 7:**
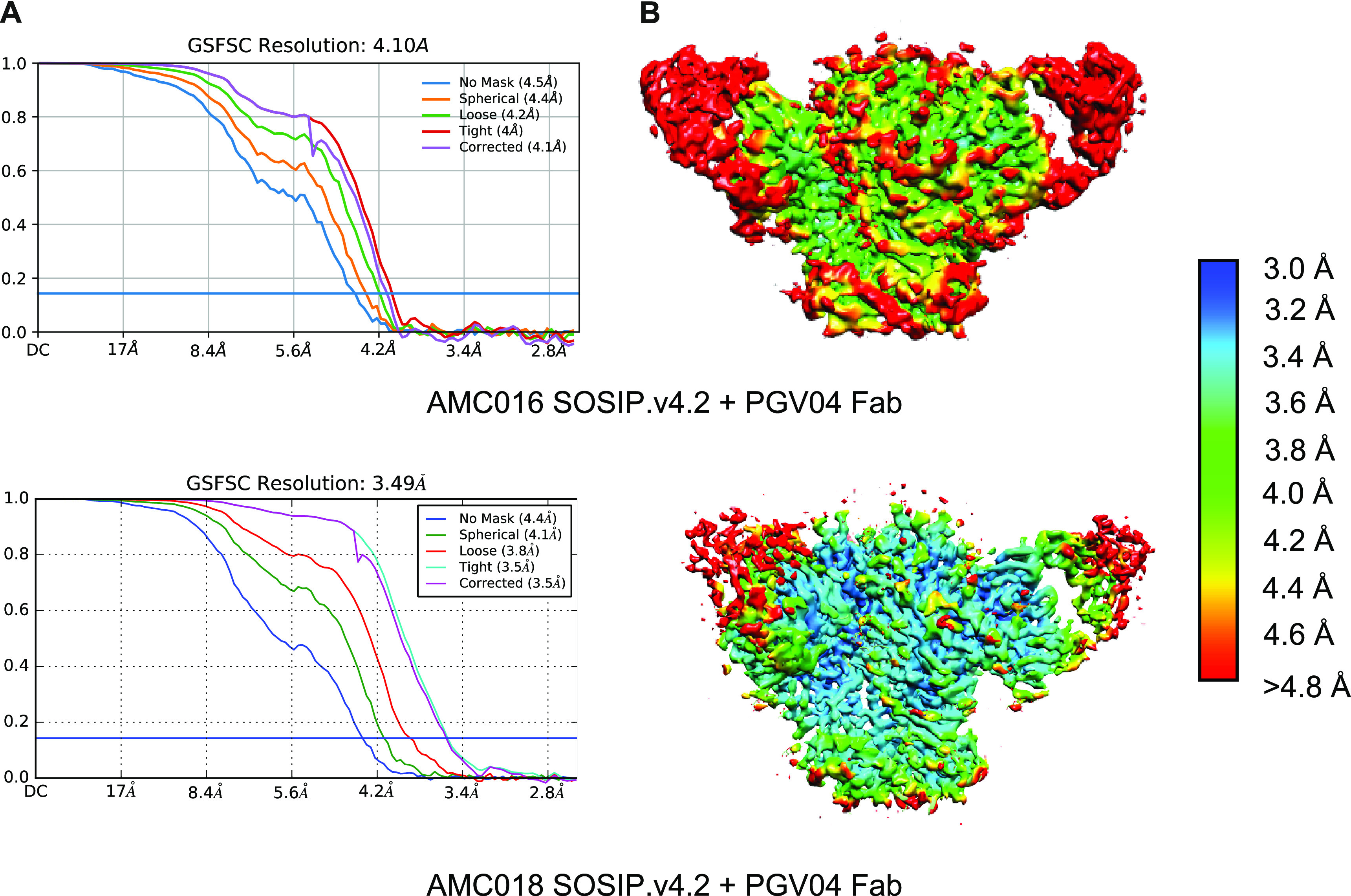
Cryo-EM parameters used for the modeling of the AMC016 SOSIP.v4.2 and AMC018 SOSIP.v4.2 trimers, complexed with PGV04 Fab. (A) Fourier shell correlation curves calculated in cryoSPARC during final refinement. (B) Local resolution maps. Colors represent the resolution (3.0 Å to > 4.8 Å).

The cryo-EM structures of the AMC016 and AMC018 trimers were solved at 4.1-Å and 3.5 Å-resolution, respectively, before they were used as immunogens (EMD-24676 and PDB ID 7RSO for AMC016; EMD-24675 and PDB ID 7RSN for AMC018). The structures of both trimers were solved when complexed with the CD4bs-directed bNAb PGV04 (PDB ID 6CRQ) (Fig. 6A and B, Fig. 7, and Table 2) (28). Overlays of the structures of the AMC016 and AMC018 trimers with that of the BG505 SOSIP.664 trimer (PDB ID 4ZMJ) showed that all three trimers are highly similar (Fig. 6C) (29). The Cα root-mean-square deviation (RMSD) value, a quantitative measure for similarity between superimposed structures, was 1.1 Å for AMC016 versus BG505 in the gp120 subunit and 1.3 Å in the gp41 subunit, while the RMSD values for AMC018 versus BG505 were 1.0 Å in gp120 and 1.5 Å in gp41.

**TABLE 2:** Cryo-EM parameters of the AMC016 and AMC018 trimers

Parameter	Analyzed trimers	
	AMC018 SOSIP.v4.2 + PGV04 Fab	AMC016 SOSIP.v4.2 + PGV04 Fab
Microscope	FEI Titan Krios	FEI Titan Krios
Voltage (kV)	300	300
Detector	Gatan K2 Sumit	Gatan K2 Sumit
Recording mode	Counting	Counting
Magnification	22.500	22.500
Moive micrograph pixel size (Å)	1.31	1.31
Dose rate {e-/[(camera pixel) × s]}	6.52	9.81
No. of frames per movie micrograph	35	35
Frame exposure time (ms)	200	200
Movie micrograph exposure time (s)	7	7
Total dose (e-/Å^2^)	26.6	40
Defocus range (μm)	−1.0 to −3.9	−1.0 to −4.0
No. of movie micrographs	3,916	3,801
No. of molecular projection images in map	150,333	77,068
Symmetry	C3	C3
Map resolution (FSC 0.143)	3.49	4.10
Map sharpening B-factor (Å^2^)	−151.9	−205.3
No. of atoms in deposited model	19,623	20,574
MolProbity score	0.95	0.72
Clashscore	0.41	0.67
EMRinger score	2.83	1.97
Privateer	Pass	Pass
pdb-care	Pass	Pass
EMD	EMD-24675^*a*^	EMDB-24676^*a*^
PDB ID	7RSN	7RSO

a Accession numbers EMD-24675 and EMD-24676 can be found at https://www.ebi.ac.uk/emdb/EMD-24675 and https://www.ebi.ac.uk/emdb/EMD-24676, respectively.

The electron density for the AMC016 structure was sufficient to allow the building of 26 glycans out of 30 PNGS (Fig. 6D, glycans indicated in green), while we were able to build 16 glycans on the AMC018 structure out of 30 PNGS (Fig. 6D, glycans indicated in green). As the overall resolution increases, electron density corresponding to dynamic or flexible regions (e.g., uncoordinated N-linked glycans) becomes more diffuse, which prevents accurate model building of those regions. Thus, although the resolution of the AMC018 trimer structure was higher than that of the AMC016 trimer structure, the reduced electron density corresponding to PNGS in the AMC018 structure reduced the number of glycans that could be built.

**FIG 8:**
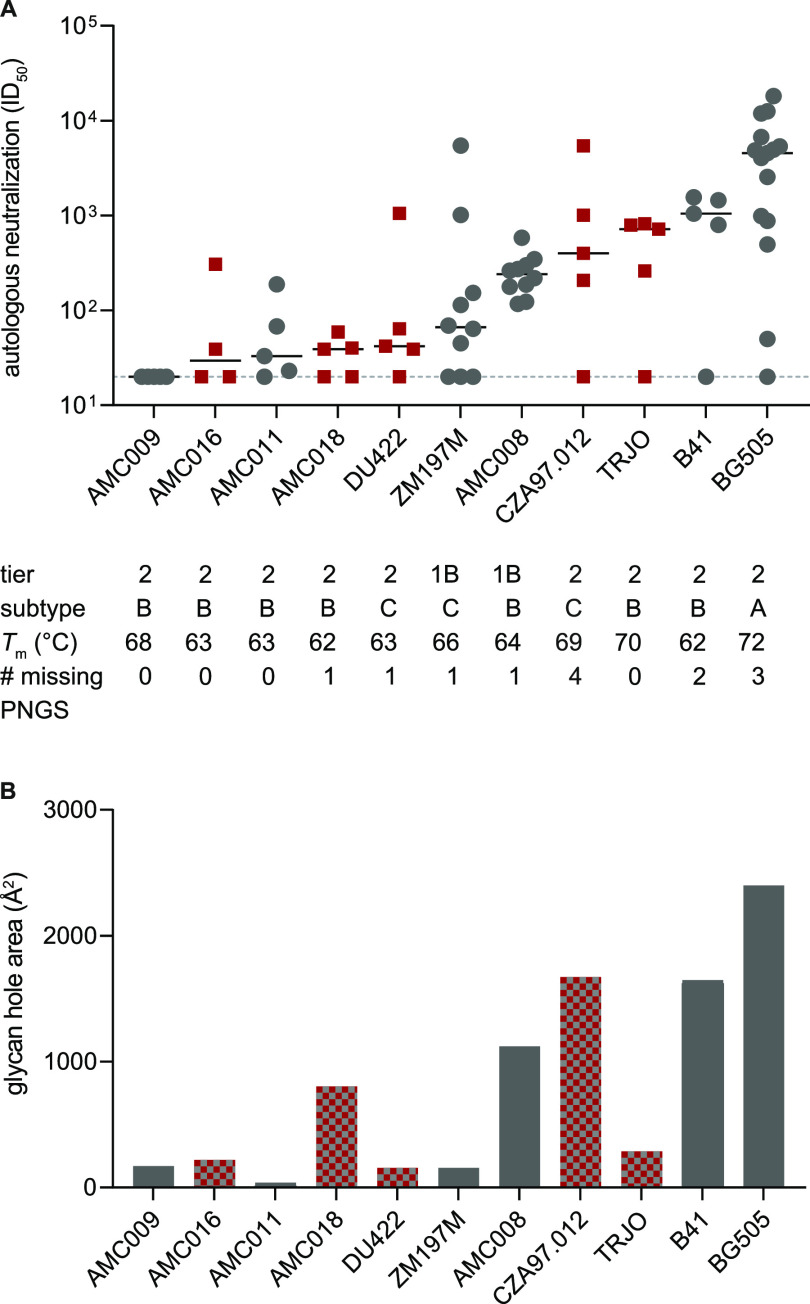
Autologous neutralization of a panel of SOSIP trimers with different characteristics. (A) The autologous NAb response at week 22 of rabbits immunized with SOSIP trimers from subtypes A, B, and C. The trimers lack zero to four conserved PNGSs. Gray spheres, SOSIP trimers with previously published immunogenicity (*n* ≥ 5). Red squares, newly analyzed SOSIP trimers of study C0048-15 and PA0064-16 (*n* = 5; *n* = 4 for the AMC016 group) (see Materials and Methods for details) (2, 3, 27). The black line indicates the median ID_50_; the dashed line represents the lower assay cutoff ID_50_ value of 20. Listed are the tier categorization, genetic subtype, *T*_m_, and the number of missing conserved PNGSs. (B) Analysis of the overall glycan hole area, using the glycan shield mapping tool (21). The color code used is the same as in panel A. (A and B) SOSIP trimers are ordered based on the median ID_50_ values, from lowest to highest. Individual values are shown in Supplemental file 2.

The AMC016 and AMC18 viruses were categorized as tier 2 (Table 3). The corresponding AMC016 and AMC018 trimers were then tested as immunogens, as were the previously described DU422, CZA97.012, and TRJO trimers (see Materials and Methods; Supplemental file 2) (18, 30, 31). In both studies, rabbits (*n* = 5) were immunized at week 0, 4 and 20, with Iscomatrix used as the adjuvant. The median autologous NAb ID_50_ values measured at week 22 were 30, 39, 42, 401, and 719 for AMC016, AMC018, DU422, CZA97.012, and TRJO, respectively (Fig. 8A, red squares; Supplemental file 2). The previously reported median ID_50_ values for AMC009, AMC011, ZM197M, AMC008, B41, and BG505 are 20, 33, 67, 240, 1,048, and 4,561, respectively (Fig. 8A, gray spheres; Supplemental file 2) (2, 3, 27).

**TABLE 3:** Tier categorization of the AMC016 and AMC018 viruses

Serum/antibody	ID_50_ (dilution) by virus^*a*^	
	AMC016	AMC018
Serum pool^*b*^		
CHAVI-0406 pool	10	10
CHAVI-0060 pool	20	49
CHAVI-0642 pool	10	22
CHAVI-0293 pool	22	118
CHAVI-0598 pool	23	125
CHAVI-0585 pool	90	288
GM ID_50_	21	60
Antibodies		
VRC01	0.16	0.27
3BNC117	0.06	0.05
CH31	0.25	0.11
CH01	>25	>25
PG9	3.9	>5
PG16	3.4	>5
10-1074	0.08	0.06
PGT128	0.16	0.04
PGT121	0.14	0.13
PGT151	0.02	0.02
2F5	11	4.7
4E10	24	8.5
10E8	2.2	0.9
CH01-31	0.47	0.21
Classification	Tier 2	Tier 2

a The two subtype B viruses were tested against serum pools and a panel of bNAbs to assess neutralization sensitivity.

b Lack of neutralization at 1:20 is represented as a value of 10. The reciprocal geometric mean (GM) ID_50_ is the 50% inhibitory concentration. The TZM-bl cell assays were performed at Duke University Medical Center (DUMC) (see Materials and Methods).

Autologous NAb titers could be influenced by various factors. Holes in the glycan shield, created by the absence of conserved PNGS, have been shown to promote autologous NAb induction (17, 26). We found a positive correlation between the number of missing PNGS and the median autologous NAb titers (Spearman *r* = 0.6913; 95% confidence interval [CI] = 0.1361 to 0.9160; *P* = 0.022). When inspecting the data, however, we noted substantial differences in the autologous NAb responses of trimers that each had the same number of missing PNGS (e.g., TRJO versus AMC009) (see Fig. 8A).

### The glycan hole area correlates with autologous neutralization.

The above analysis suggested that the number of missing PNGS affects autologous neutralization but also that other factors may be relevant. The Los Alamos glycan shield mapping tool (from here on abbreviated glycan shield mapping tool) allows for a more accurate prediction of the overall glycan hole area than just the number of missing PNGS (21). The tool takes the 3D structure of the Env trimer into account, as well as the shielding effect of neighboring glycans, assuming a radius of 10 Å for each glycan. Regions that are never shielded by glycans, such as the gp120-gp41 interface, the CD4 binding-site (CD4bs), and the fusion peptide, are excluded from the analysis. The focus is on conserved PNGS, which are present in >50% of HIV-1 group M viruses, and it is assumed that they are occupied fully.

We analyzed the above 11 SOSIP trimers using the glycan shield mapping tool. The predicted glycan hole areas varied among the trimers (Fig. 8B; Supplemental file 2). The areas were relatively small (<200 Å^2^) for AMC011, AMC009, DU422, and ZM197M (33, 170, 156, and 156 Å^2^); intermediate (<200 to 1,000 Å^2^) for AMC016, TRJO, and AMC018 (218, 286, and 803 Å^2^); and large (>1,000 Å^2^) for AMC008, B41, CZA97.012, and BG505 (1,120, 1,641, 1,671, and 2,401 Å^2^). We found a positive correlation between the glycan hole area and the median autologous NAb titers (Spearman *r* = 0.7062; 95% CI = 0.1645 to 0.9206; *P* = 0.019).

**FIG 9:**
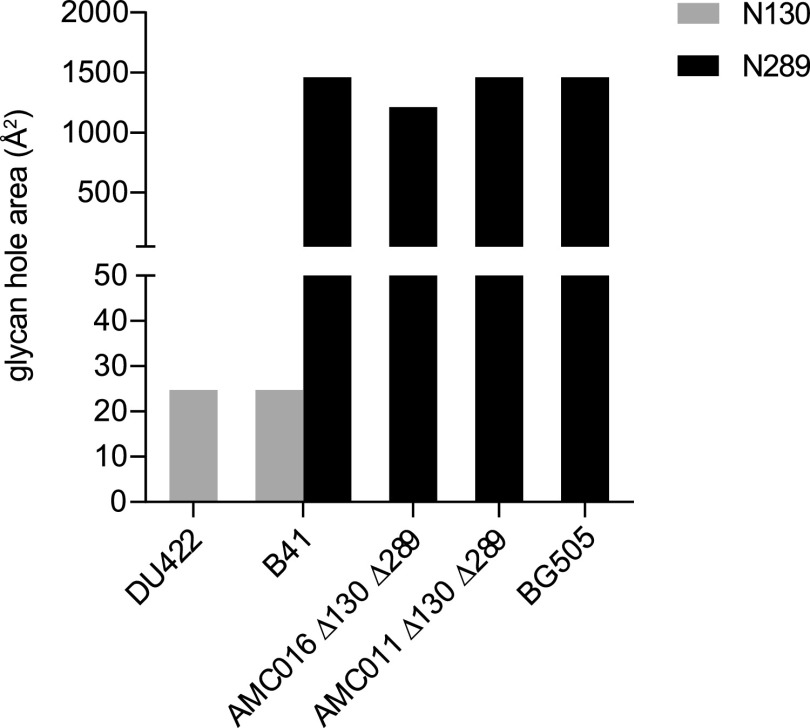
The size of the glycan hole area created by the absence of N130 and N289. The SOSIP trimers from Fig. 8 that lack one or both glycans, either naturally or via knockout, were analyzed with the glycan shield mapping tool (21). For some trimers, the missing N130 glycan did not create a glycan hole. SOSIP trimers are ordered based on the median ID_50_ values, from lowest to highest.

The tool also allows us to assess the individual surface area that is exposed by the absence of a conserved PNGS. The differences between the contributions to the overall glycan hole area made by N130 and N289 were analyzed, based on trimers that miss the two PNGS either naturally or by design. The absence of the N130 PNGS from DU422, CZA97.012, AMC011 Δ130Δ289, AMC016 Δ130Δ289, and BG505 trimers did not generate a glycan hole but created a very small hole on DU422 and B41 trimers (24.8 Å^2^ for both) (Fig. 9, light gray). In contrast, the absence of N289 PNGS created a large glycan hole (1,461 Å^2^ for B41, AMC011 Δ130Δ289, and BG505; and 1,211 Å^2^ for AMC016 Δ130Δ289) (Fig. 9, dark gray). The above estimations are consistent with our finding that the hole created at N289 strongly influenced how autologous NAbs were induced by the AMC011 Δ130Δ289 and AMC016 Δ130Δ289 trimers (see Fig. 5C). They are also consistent with observations that the N130 site and the nearby region are not immunogenic on B41 and BG505 trimers (17, 18, 26, 32). However, as the autologous NAb titers were analyzed in detail only for the B41 and BG505 trimers, it is possible that the N130 glycan hole may be immunogenic on other trimers.

### The glycan hole area is a predictor for the induction of autologous neutralization.

Factors other than the number of missing PNGS and glycan hole area that might affect the induction of autologous NAbs are best studied in combination to supplement the simple correlation analyses described above. These possibly contributory factors include the neutralization tier categorization and genetic subtype of the trimer immunogen and the corresponding test virus (see Supplemental file 2). Trimer stability, for which *T*_m_ values are a surrogate, may also be relevant, as immunogenic, non-neutralization epitopes become accessible if trimers dissociate into dimers and monomers.

**FIG 10:**
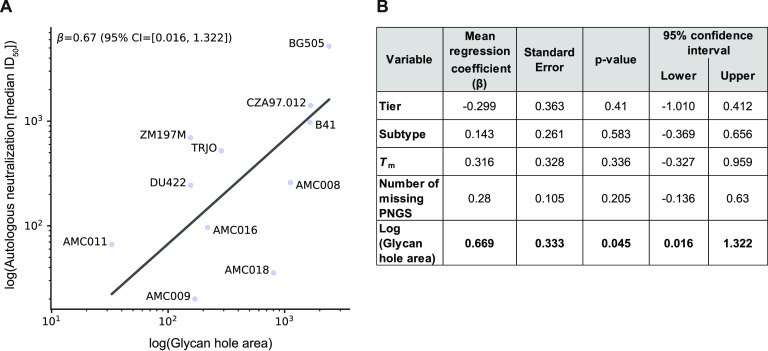
Fitted correlation based on the mean regression coefficient of log glycan hole area and log autologous neutralization. Tier, subtype, *T*_m_, and number of missing PNGSs were assumed to not have any effect. (A) Data points are the median log autologous neutralization values for each trimer, across all animals. The linear regression coefficient (β) is indicated. (B) Coefficients of predictor variables in the generalized linear model.

We performed a linear regression analysis to study the above factors (Fig. 10A). The analysis was based on the median ID_50_ values for the 11 SOSIP trimers shown in Fig. 8 (for individual values, see Supplemental file 2). The log values of the autologous neutralization data were fitted to a generalized linear model that included the following predictor variables: neutralization tier categorization, genetic subtype, *T*_m_, number of missing PNGS, and log glycan hole area. The estimated 95% confidence intervals of the regression coefficients for tier, subtype, *T*_m_, and the number of missing PNGS included zero (Fig. 10B). Hence, these four predictors are unlikely to influence autologous neutralization. In contrast, the mean regression coefficient of the glycan hole area was 0.67, suggesting a positive correlation. Although the estimated 95% confidence interval for glycan hole area is above zero, the interval is relatively wide, with a lower bound near zero (95% CI = 0.016 to 1.322). Hence, there is a degree of uncertainty about the influence of glycan hole area on autologous neutralization.

## DISCUSSION

We establish here that the area of glycan holes, created when conserved PNGS are missing, influences the induction of autologous NAbs by native-like HIV-1 SOSIP trimers. We also describe here a new SOSIP trimer, AMC016 SOSIP.v4.2, as well as high-resolution cryo-EM structures for the AMC16 and AMC018 trimers, adding to the repertoire of stable and native-like Env subtype B trimers.

The engineered knockout of the PNGS at N130 and N289 had subtle impacts on glycan composition and occupancy. The changes were localized mostly on the trimer apex, near N130, and in the oligomannose patch, near N289. The reduction in Man_9_ content of surrounding glycans suggests that the accessibility of ER α-mannosidase is increased when a PNGS is removed (16). Greater access to oligosaccharyltransferase (OST) might account for the increased occupancy of the N141 site at the trimer apex. The slightly reduced occupancy of N136 for AMC011 Δ130Δ289 is harder to explain, although it is known that OST can sometimes skip over one or more PNGS when several of them are located close together in the primary sequence (23). Our data show how the same glycan deletion on SOSIP trimers with similar characteristics can have a similar effect on the overall glycan shield but different effects at the site-specific level. The individual *env* sequence as well as how the trimer folds (i.e., its structure and conformation) all seem to play a role.

The engineered or natural absence of a conserved PNGS creates glycan holes of different sizes on different SOSIP trimers. Deleting N130 does not create a measurable glycan hole or yields only a very small one, as was reported previously for the BG505 SOSIP trimer (21). The structure and dense local glycosylation of the V1 loop and the occupancy and composition of the neighboring glycans probably work together to create redundancy in that region of the glycan shield. Conversely, the absence of the N289 PNGS created a large glycan hole on all the SOSIP trimers we studied. That observation is consistent with how the autologous NAb response in animals immunized with B41 and BG505 SOSIP trimers is dominated by antibodies against the N289 glycan hole and not its N130 counterpart (18). Where a missing PNGS is located within the overall glycan network matters from the perspective of inducing autologous NAbs.

We found that the glycan hole area is correlated positively with the autologous neutralization titer. Furthermore, the glycan hole area is a better predictor for NAb induction than the number of missing PNGS, although the two factors are clearly related. This relationship highlights the importance of missing PNGS at sites that are not conserved, as the resulting glycan holes can create immunodominant epitopes for narrow-specificity NAbs. However, our analyses were unable to explain all the variation in the NAb response. An explanation might be the variation in how autologous NAbs are induced. The inherent immunogenicity of the lining and base of a glycan hole and how accessible it is, the extent to which the compactness of a trimer affects epitope access more generally, the incomplete occupancy of various PNGS, and other factors are probably also relevant to various and not readily quantifiable extents.

Our analysis has limitations. First, the glycan shield mapping tool assumes full occupancy, which is not the case for every PNGS on every trimer (33). A NxS motif is less likely to be glycosylated than NxT, and PNGS near the trimer base are generally relatively underoccupied (3, 23). Second, PNGS underoccupancy is more pronounced on soluble, recombinant trimers than on virus-associated Env (34, 35). Hence, antibodies induced by underoccupied PNGS are not detected in neutralization assays, except when tested against a mutant virus from which the PNGS is knocked out (23, 34). An example is the N611-directed antibodies induced by BG505 SOSIP trimers on which N611 is underoccupied (23, 36). Third, antibodies elicited by knocking out conserved glycans were assumed to be able to cross-neutralize viruses that miss the same glycans. However, our neutralization data with B41 indicates that this is not always the case. These data are in line with the finding that SOSIP trimers, sharing the same glycan holes, can induce distinct autologous NAbs (26).

Nevertheless, the data presented here can guide the identification and assessment of potential new native-like trimer vaccine candidates and facilitate the selection of *env* sequences with certain qualities, such as a complete glycan shield or the presence of specific glycan holes. Our findings highlight the possibility of modifying trimers by creating glycan holes and focusing the immune response on desired epitopes. This strategy can be used in prime-boost strategies, for example as components of germline-targeting approaches (37–39). For example, precursor VRC01 B cells can be activated using germline-targeting immunogens that lack glycans around the CD4bs (39). Subsequent “shaping” and “polishing” iimmunogens have gradually more complete glycan shields to mature responses to recognize the epitopes in the context of glycans.

## MATERIALS AND METHODS

### Design and expression of Env SOSIP trimers.

The parental SOSIP trimers AMC011 SOSIP.v5.2 and AMC016 SOSIP.v4.2 were derived from subtype B virus-infected participants of the ACS on HIV/AIDS who enrolled in the men having sex with men (MSM) cohort (40). The design and characterization of AMC011 SOSIP.v5.2 have been described elsewhere, while the AMC016 SOSIP.v4.2 trimer is described below (3). N130 and N289 were knocked out from both SOSIP trimers by site-directed mutagenesis (QuikChange II kit; Agilent Technologies) as described (5, 41). The mutated amino acids are indicated in Fig. 2A. Both trimers were expressed by transient transfection in HEK-293F cells and purified by PGT145 affinity chromatography (2, 5). The design and characteristics of the following SOSIP trimers used in Fig. 8 were published previously: AMC009 SOSIP.v5.2 (3), AMC008 SOSIP.v4.2 (2), B41 SOSIP.v4.1 (25), BG505 SOSIP.v4.2 and v5.2 (2, 42), CZA97.012 SOSIP.664 (31), DU422 SOSIP.664 (30), and ZM197M SOSIP.v4.2 and v5.2 (42). TRJO SOSIP.v5.2 will be described in a future manuscript (unpublished data). The AMC016 SOSIP.v4.2 trimer was based on an *env* sequence that was isolated at month 9 postseroconversion from individual H19974, who did not develop bNAbs. The *env* sequence used to generate the AMC018 SOSIP.v4.2 trimer was isolated at month 3 postseroconversion from individual H19961, who also did not develop bNAbs. The genes encoding the AMC016 and AMC018 SOSIP.v4.2 constructs were designed as described previously (2, 5). The codon-optimized *env* genes were obtained from GenScript (Piscataway, NJ), cloned into the pPPI4 expression vector, expressed in HEK-293F cells, and affinity purified with the bNAb PGT145 (2, 5). A D7324 epitope-tag sequence (GSAPTKAKRRVVQREKR) was introduced to the AMC016 SOSIP.v4.2 sequence, C-terminally of residue 664 in gp41_ECTO_, to allow analysis in a D7324-MAb-capture ELISA and DSC.

### Blue native-PAGE and SDS-PAGE.

SOSIP trimers were analyzed on blue native-PAGE and SDS-PAGE gels to check trimerization and cleavage by furin (5).

### D7324-capture ELISA.

This ELISA for characterizing PGT145-purified SOSIP trimer was performed as described previously (5). Briefly, Microlon 96-wells plates (Greiner Bio-One, Alphen aan den Rijn, The Netherlands) were coated overnight with sheep polyclonal antibody D7324 (Aalto Bioreagents, Dublin, Ireland) at 10 μg/ml. Purified D7324-tagged SOSIP trimers (2.75 μg/ml) were captured on the plate, and the binding of a panel of bNAbs and non-neutralizing antibody 17b was tested. Goat anti-human horseradish peroxidase (HRP)-labeled IgG was used as a secondary antibody.

### Differential scanning calorimetry.

Thermal denaturation was probed with a nano-DSC calorimeter (TA Instruments, Etten-Leur, The Netherlands), and a two-state scaled model was applied to determine the thermal denaturation (2). DSC experiments were performed with the D7324-tagged SOSIP protein; the presence of the D7324-tag does not influence *T*_m_ values (2).

### Negative-stain electron microscopy and image processing.

The imaging and processing of the SOSIP trimers were described previously (2).

### N-glycan profiling using HILIC-UPLC.

N-linked glycan profiling using HILIC-UPLC was described in detail previously (13, 23). N-linked glycans were released from trimers in-gel by digestion with PNGase F (New England BioLabs). The released glycans were fluorescently labeled with procainamide and analyzed with a Glycan BEH amide column (2.1 mm by 100 mm, 1.7 mM; Waters) in a Waters Acquity H-Class UPLC instrument, and the fluorescence was measured. To determine the relative abundance of oligomannose-type glycans, labeled glycans were digested for 16 h at 37°C with endoglycosidase H (Endo H; New England BioLabs). The digested glycans were purified on a polyvinylidene difluoride (PVDF) protein-binding membrane plate (Millipore) and then analyzed.

### Site-specific glycan analysis using mass spectrometry.

N-linked glycan composition and occupancy at every present PNGS was analyzed with mass spectrometry, as described previously (23). Some of the PNGS frequently present low intensity glycoproteins. In order to still obtain information on these sites, the glycans that are present on the glycopeptides were homogenized to boost the intensity of these peptides (23). In this way, the ratios of oligomannose glycans/complex glycans/unoccupied PNGS can be determined, but fine processing information is lost.

### Single particle cryo-electron microscopy.

The AMC016 SOSIP.v4.2 and AMC018 SOSIP.v4.2 trimers were incubated with the PGV04 Fab at a 2-fold molar excess of Fab/protomer, overnight at room temperature. The complexes were purified using a Superose 6 10/300 column (GE health care) in Tris-buffered saline (TBS) to remove unbound Fab. The purified complexes were mixed with *N*-dodecyl-d-maltoside to a final concentration of 675 μM and applied to C-Flat grids (CF-2/2-4C; Electron Microscopy Sciences, Protochips, Inc.). The grid had been plasma cleaned for 5 s using a mixture of Ar/O2 (Gatan Solarus 950 plasma system). Samples were manually blotted using filter paper and then immediately plunged into liquid ethane using a manual freeze plunger. Data were collected via the Leginon interface on an FEI Titan Krios operating at 300 keV mounted with a Gatan K2 direct electron detector in counting mode at 22,500 × nominal magnification resulting in a calibrated pixel size of 1.31 Å/pix at the objective level (43). Dose rate and additional data collection parameters are reported in Table 2. Movies were imported into cryoSPARC v2, and frames were aligned using full-frame motion correction (44). The contrast transfer function (CTF) for each aligned micrograph was estimated using Gctf (45). The HIV Env portion of the BG505 SOSIP.664 trimer (PDB ID 5ACO) was converted to an EM density and low pass filtered to 40 Å using pdb2mrc and subsequently used as a template for particle picking within cryoSPARC v2 (44, 46, 47). 2D classification, Ab-initio 3D reconstruction, homogenous 3D refinement, and local motion correction were conducted with cryoSPARC v2 (44). Per-particle CTF estimation was conducted using Gctf (45). Local-resolution maps were generated using cryoSPARC v2 (44). Initial molecular models for the AMC016 SOSIP.v4.2 and AMC018 SOSIP.v4.2 trimer were generated using the Modeller homology modeling plug-in UCSF Chimera (48, 49). The templates for AMC016 SOSIP.v4.2 were the JR-FL Env structures (PDB ID 5FYK [gp120] and PDB ID 5FUU [gp41]) (9, 50). The template for AMC018 SOSIP.v4.2 was the AMC009 SOSIP.v4.2 Env structure (PDB ID 6VO3) (3). Models were docked into the corresponding EM density map along with the PGV04 Fv (PDB ID 6CRQ) using UCSF Chimera (49). Regions not supported by density were removed, and N-linked glycans were added using Coot (51). The models were iteratively refined into the EM density maps using RosettaRelax and Coot (52, 53). Glycan structures were validated using Privateer, and the overall structures were evaluated using EMRinger and MolProbity (54–56).

### Rabbit immunizations.

Rabbit immunizations with AMC016 SOSIP.v4.2, AMC016 SOSIP.v4.2 Δ130Δ289, AMC011 SOSIP.v5.2, AMC011 SOSIP.v5.2 Δ130Δ289, and B41 SOSIP.v4.1 trimers were carried out under approval number C0026-17, under subcontract at Covance (Denver, PA, USA). Female New Zealand white rabbits (5 per group) were immunized intramuscularly with 30 μg of SOSIP trimer at weeks 0, 4, and 20, with the GLA-LSQ adjuvant (IDRI, Seattle, WA; obtained via the Bill and Melinda Gates Foundation (BMGF) collaborative network). The B41 trimer experiment has been published, but the sera were reanalyzed alongside other sera from same study to generate comparable data (see Fig. 3) (19). We learned *post hoc* that GLA-LSQ is an inefficient adjuvant, such that NAb titers are lower in this experiment than in previously published experiments where a different adjuvant was used (2, 3). Sera from week 22 in the present (GLA-LSQ) experiment were used to derive the autologous NAb responses as plotted in Fig. 5, but they were excluded from the analysis in Fig. 8 and 10 for a noncomparability reason. All of the week 22 NAb titer data shown in Fig. 8 and 10 were derived from experiments in which the adjuvant was Iscomatrix. NAb responses to the AMC009, AMC011, ZM197M, AMC008, B41, and BG505 trimers (indicated in gray spheres in Fig. 8) were published previously (2, 3, 27).

The immunogenicity of the AMC016 and AMC018 SOSIP.v4.2 trimers was assessed under approval number C0048-15 at Covance. The data are presented in Fig. 8, indicated in dark red squares, and Fig. 10. The protocol was identical to that described above, except that 22 μg of trimer was used with the Iscomatrix adjuvant (CSL Ltd., Parkville, VIC, Australia). In Covance study PA0064-16, the TRJO SOSIP.v5.2, DU422 SOSIP.664, and CZA97.012 SOSIP.664 trimers were used at 30 μg with Iscomatrix (data shown in dark red squares in Fig. 8 and 10). Serum samples of PA0064-16 were analyzed at DUMC, all other sera were analyzed at Amsterdam UMC. In both the C0048-15 and PA0064-16 studies, the autologous NAb titers were determined using week 22 sera (for individual ID_50_ values see Supplemental file 2).

### Neutralization assay and generation of infectious molecular clones.

A standard TZM-bl cell neutralization assay was used to measure the autologous NAb titers (2, 57–60). The AMC011 and AMC016 Δ289 and Δ130Δ289 viral variants were ordered as infectious molecular clones, and virus infectivity was quantified in a standard TZM-bl cell assay via titration. The deletion of the N130 and/or N289 glycans did not affect virus infectivity. The parental virus and the corresponding glycan variants neutralized VRC01 with a similar IC_50_. These data are in line with previous findings that removal of most single PNGS, including the ones studies here, do not have a major impact on infectivity (61, 62). The other viruses (DU422, CZA97.012, and TRJO) used to analyze sera from the P0064-16 study have been described previously (18, 63). The AMC016 and AMC018 viruses were tested at DUMC against serum pools and a panel of well-characterized antibodies, using a standard TZM-bl cell assay (63, 64).

### Statistical analyses.

NAb titers (ID_50_) of groups in Fig. 5 were compared by using the two-tailed Mann-Whitney *U* test. Spearman’s rank correlation coefficients and *P* values (two-tailed) were calculated to determine the correlation between median autologous NAb titers and the number of missing PNGS or the glycan hole area. Both tests were performed in GraphPad Prism 8. To analyze the data in Fig. 10, we performed a log transformation of the median autologous neutralization value of each trimer to reduce the right skewness of the data. The *T*_m_ values of the ZM197M and BG505 SOSIP.v4.2 and v.5.2 variants were averaged (*T*_m_ of 62.6°C versus 69.2°C for ZM197M variants and 69.3°C versus 75.0°C for BG505 variants, respectively). We then fitted the neutralization values (median ID_50_) ($Y_{\mathrm{neut}}$) to a generalized linear model that included predictor variables, such as tier categorization ($X_{\mathrm{tier}}$), subtype ($X_{\mathrm{subtype}}$), midpoint of thermal denaturation ($X_{T_{m}}$), number of missing PNGS ($X_{\mathrm{PNGS}}$), and log glycan hole area $\left(X_{\mathrm{glycan}}\right)$, as follows:

$$Y_{\mathrm{neut}}=\underset{i}{\sum }\beta_{i}X_{i}+c$$

where $i=\left\{\mathrm{tier},\mathrm{subtype},T_{m},\mathrm{PNGS},\mathrm{glycan}\right\}$. We encoded the categorical predictors $X_{\mathrm{subtype}}$ as subtype A = 0, subtype B = 1, and subtype C = 2; $X_{\mathrm{tier}}$ as 0 when tier = 2 and 1 when tier = 1B. Computing eigenvalues on the covariance matrix between all predictor variables, we assessed that there is no multicollinearity between them. The model was fitted using the statsmodels package in Python (65).

### Data availability.

For the following trimers, the cryo-EM reconstruction and the molecular model described here have been deposited in the Electron Microscopy Data Bank and Protein Data Bank, under the listed accession codes: AMC016 SOSIP.v4.2 (EMD-24676; PDB ID 7RSO) and AMC018 SOSIP.v4.2 (EMD-24675; PDB ID 7RSN).
